# Tuberculous Laryngitis

**Published:** 1901-03

**Authors:** 


### Tuberculous Laryngitis.

In the first stage place a dessertspoonful of the following in a pint of water kept slowly boiling and let the patient inhale:

℞ Tinct. canellæ,
Tinct. bellad.,
Tinct. opii ....................aa 30
Tinct. eucalypti ................. 100

In the second period lactic acid gives excellent results, but its application must be energetic and repeated.—BOMMIER.

When there is pain:

℞ Di-iodiform .................... 8.

Hydrochl. cocainæ .............. 0.08

Hydrochl. morphinæ ........... 0.04

Give four to eight respirations daily.

—LEDUC.

For an insufflation or gargle:

℞ Cocain. hydrochl. ............. 1.
Morphin. hydrochl. ........... 0.80
Acid. phenic ................. 1.
Antipyrin .................... 4.
Glycerini puri,
Aq. menthæ pip.............aa 50.
Aq. destil. .................... 300.

M.

—BOMMIER.

